# The role of Erzhi Tiangui formula in expected poor ovarian responders undergoing in vitro fertilization-embryo transfer: A multicenter, randomized, double-blind, placebo-controlled trial

**DOI:** 10.1097/MD.0000000000034088

**Published:** 2023-07-07

**Authors:** Qiao-Song Han, Yue Zhou, Wen Chen, Jing-Yan Song, Zhen-Gao Sun

**Affiliations:** a The First Clinical College, Shandong University of Traditional Chinese Medicine, Jinan, China; b The College of Traditional Chinese Medicine, Shandong University of Traditional Chinese Medicine, Jinan, China; c Reproductive Center of Integrated Medicine, The Affiliated Hospital of Shandong University of Traditional Chinese Medicine, Jinan, China.

**Keywords:** Chinese medicine, Erzhi Tiangui formula, IVF-ET, poor ovarian response, study protocol

## Abstract

Advanced age is one of the primary risk factors for infertility. Poor ovarian response (POR) to exogenous gonadotropin is a prominent characteristic of advanced-age women undergoing in vitro fertilization and embryo transfer (IVF-ET), which results in fewer retrieved oocytes and poor pregnancy outcomes. Traditional Chinese medicine (TCM) has been shown to improve female fertility. Erzhi Tiangui (EZTG) formula, in the form of granules with 10 herbal ingredients, demonstrated potential benefits in improving oocyte and embryo quality and ovarian reserve. Thus, this study aims to evaluate the efficacy and safety of EZTG formula.

**Method:**

The study is a multicenter, double-blind, placebo-controlled, randomized controlled trial (RCT), which will be conducted at 10 reproductive centers of tertiary hospitals. This study will enroll 480 women with expected POR of advanced age (≥35 years old) who fulfill the 2011 Bologna criteria. Participants will be assigned to either the EZTG group or the placebo group at random in an equal ratio. Each individual will receive conventional IVF-ET with EZTG granules or placebo as a complementary treatment. The primary outcome is the number of oocytes retrieved. Adverse events and safety assessments will be also conducted.

**Discussion:**

This study aims to provide robust evidence of the efficacy and safety of EZTG formula as a complementary treatment for advanced-age women with expected POR undergoing IVF-ET.

## 1. Introduction

The postponement of childbearing has become a global trend, which led to a gradual increase in the proportion of infertile women of advanced age. In women older than 35 years old, ovarian reserve and oocyte quality are diminished, making assisted reproductive technology (ART) more likely to be required to achieve pregnancy.^[1–3]^ For women undergoing in vitro fertilization-embryo transfer (IVF-ET), POR to controlled ovarian stimulation (COS) remains a serious dilemma. POR indicates a reduction in follicular response, resulting in a reduced number of retrieved oocytes. And advanced age is considered the most relevant risk factor for POR.^[4]^ POR is generally associated with poor clinical outcomes, including fewer retrieved oocytes, a lower clinical pregnancy rate, and a lower cumulative live birth rate (CLBR),^[5–7]^ which was referred to as “expected poor responders” or “low prognosis patients.”

Several adjuvant treatments before or during IVF-ET have been suggested for the population, such as dehydroepiandrosterone, estradiol, and growth hormone supplementation.^[8–10]^ Nonetheless, the evidence of efficacy is still limited and not satisfying.^[11]^ Traditional Chinese medicine (TCM) is an independent medical system with a rich theoretical basis. According to TCM theory, the “kidney,” also known as “Shen,” is in charge of the body aging and reproductive abilities. The kidney qi and kidney yin can be abstractly summarized as the ability to maintain the functioning of the female reproductive system and the reserve of female fertility, respectively. Therefore, the deficiency of kidney qi and kidney yin will accelerate the aging process and aggravate the loss of fertility, which is analogous to the pathogenesis of diminished ovarian reserve, and POR during IVF. Notably, some studies have indicated the benefit of kidney-tonifying TCM in improving the prognosis of elderly women with POR.^[12–14]^

Erzhi Tiangui (EZTG) formula, with the function of tonifying the kidney-qi and nourishing the kidney-yin, is in the form of granules with ten herbal ingredients. It has been widely applied in reproductive centers for over 20 years and demonstrated benefits as a supplementary treatment during ART and IVF for advanced-age women with expected POR. Some previous studies have indicated the benefits of EZTG on the clinical outcome in the population and explored the mechanism by which EZTG improve the ovarian reserve and quality of oocytes and embryos.^[15–19]^

The complementary and synergistic role of TCM for individuals undergoing IVF is an area that merits further attention and research.^[20,21]^ The present study is a multi-center, double-blinded, placebo-controlled, large-scale, superiority randomized controlled trial (RCT) with a robust methodological design. The purpose of the study is to provide robust clinical evidence that EZTG formula are effective in improving oocyte yield and other clinical outcomes in advanced-age women with expected POR undergoing IVF-ET.

## 2. Methods and analysis

### 2.1. Study design

**Figure 1.**
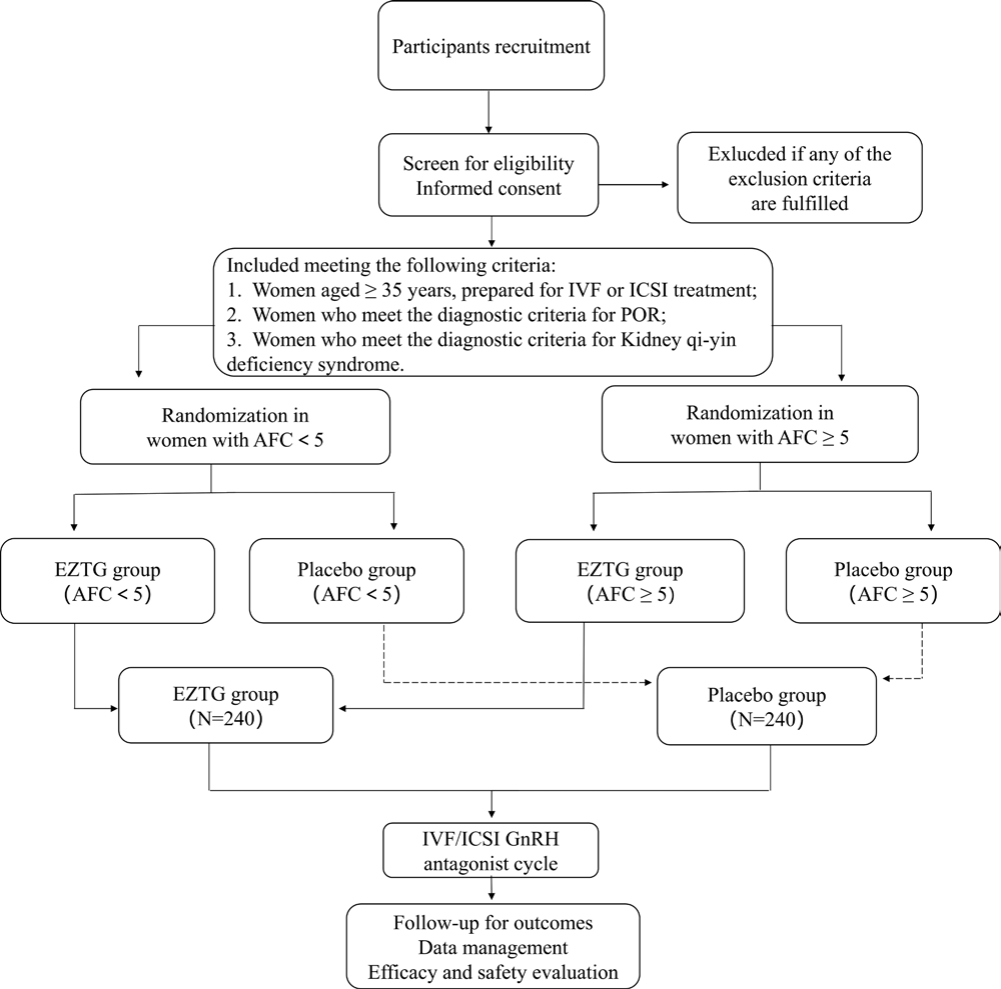
Flow diagram of participant recruitment (AFC = antral follicle count, EZTG = Erzhi Tiangui, GnRH = gonadotrophin releasing hormone, ICSI = Intracytoplasmic sperm injection, IVF = in vitro fertilization, POR = poor ovarian response).

The study is designed as a multi-center, prospective, randomized, double-blinded, placebo-controlled trial. We recruited participants who are scheduled IVF-ET from reproductive centers in ten tertiary hospitals. The primary study site is the Reproductive Center of Integrated Medicine of the Affiliated Hospital of the Shandong University of TCM. Patient enrollment is scheduled from May 2023 to June 2025. To ensure the consistency of the procedures in all the centers, All participating researchers will be uniformly trained. Regular communication and study visits will be performed at all study sites. In addition, meetings with all study sites will be held annually or more frequently. The study protocol is designed and written based on the SPIRIT checklist.^[22]^ The trial was registered at the National Institutes of Health clinical trials database (ClinicalTrials.gov, ID: NCT05698550). The study flowchart is shown in Figure 1, and the timetable of the study is summarized in Table 1.

**Table 1:**
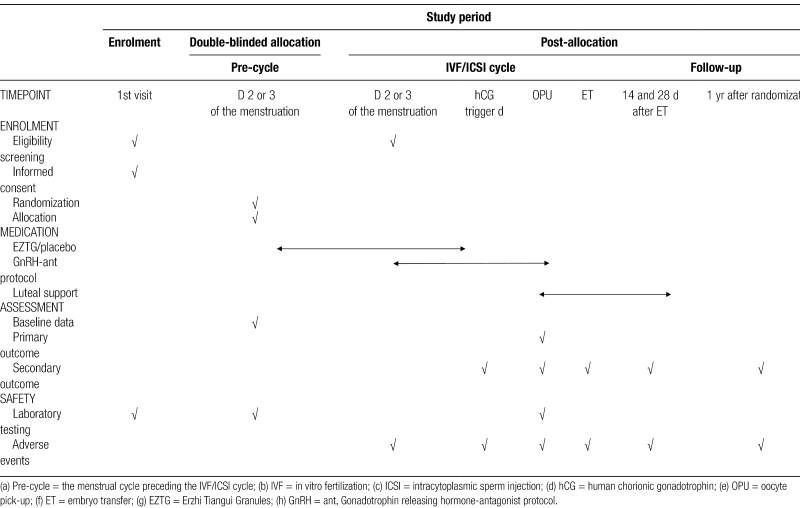
Timetable of the study.

### 2.2. Patients recruitment

A total of 480 aged women diagnosed with POR will be included in the study. Potentially eligible patients will be recruited by the clinicians in the reproductive center of ten tertiary hospitals. The detail about the trial will be introduced to them on their first visit. After a wash-out period of at least 2 weeks, eligible women will be arranged a second visit on day 2 or 3 of a menstrual cycle. Then, all couples will provide written informed consent before participation following the approval by the ethics committees of participating reproductive centers. Given the high prevalence of the disease and the large outpatient volume in participating centers, we will not apply other methods of recruitment.

### 2.3. Patient and public involvement

The study protocol was designed without patient or public participation. The randomization, allocation, and treatment procedures will not be interfered with by participants’ preferences. All results of the study will be distributed to participants upon request.

### 2.4. Eligibility criteria

#### 2.4.1. Diagnostic criteria of POR.

The diagnostic criteria of POR are following the ESHRE Bologna criteria,^[4]^ which included: advanced age (>40 years) or any other risk factor for a POR; retrieval of <3 oocytes following previous conventional ovarian stimulation; and reduced ovarian reserve test (antral follicle count [AFC] < 5–7 or anti-Müllerian hormone < 0.5–1.1 ng/mL). Patients who meet at least the above 2 conditions will be diagnosed with POR.

#### 2.4.2. Diagnostic criteria for TCM kidney qi-yin deficiency syndrome differentiation.

Kidney qi-yin deficiency syndrome is associated with the following symptoms:

(1) Heavy menstrual bleeding (>80 mL) or light menstrual bleeding (<5 mL), along with the color of dark red or thin and watery blood; (2) The waist and knees are sour and weak, or pain in the heels; (3) Dizziness, tinnitus, and mental exhaustion; (4) Low sexual desire, less vaginal discharge, and loss of armpit or pubic hair; (5) Typical pulse manifestation: deep and weak pulse in both wrists. The diagnosis of kidney qi-yin deficiency is made if there are 3 or more of the above symptoms, while "Heavy menstrual bleeding (>80 mL) or light menstrual bleeding (<5 mL), along with the color of dark red or thin and watery blood" and "The waist and knees are sour and weak, or pain in the heels" are necessary conditions to fulfill. The syndrome identification will be performed by 2 associate professors independently examining each patient. Participants will be included if the syndrome diagnosis is consistent from both professors.

#### 2.4.3. Inclusion criteria.

1. Women aged ≥35 years, eligible for IVF or Intracytoplasmic sperm injection treatment.
2. Women who meet the diagnostic criteria for POR.
3. Women who meet the diagnostic criteria for kidney qi-yin deficiency syndrome.

#### 2.4.4. Exclusion criteria.

Women with any of the following exclusion criteria are not eligible to participate in the study:

1. Women aged ≥ 43 years.
2. Women with body mass index ≥35 kg/m^2^.
3. Women undergoing oocyte donor cycle.
4. Women with a history of recurrent spontaneous abortion or repeated implantation failure, defined as 2 or more spontaneous abortions or failed IVF-ET attempts.
5. Women undergoing a cycle of In vitro Maturation or Preimplantation Genetic Testing.
6. Women with uterine abnormalities, congenital (e.g., bicornuate uterus) or acquired (e.g., severe adenomyosis and multiple endometrial polyps).
7. Women with uterine appendage abnormalities (e.g., non-surgically treated hydrosalpinx and ovarian endometriosis cyst requiring surgery).
8. Unilateral ovarian absence.
9. Women allergic to or intolerant of EZTG or other medications applied in the study.

### 2.5. Dropout and discontinuation criteria

Patients who withdraw from the study voluntarily, who experience poor compliance during the study period, or who are considered not suitable for further participation in the trial by the researcher will be removed from the RCT. The reasons include, but are not limited to the following: Participants do not take EZTG at the prescribed dosage or duration; Participants experience adverse reactions or complications related to EZTG during the study period; Participants are pregnant before IVF cycle; Participants take other medications during the study that may interfere with the study results, such as oral conceptive pills and estradiol. The concurrent use of other TCM preparations will also not be allowed.

The trial will be terminated if there are any of the following conditions: The double-blind design is accidentally disclosed or broken, leading to the failure of the trial; Existing evidence to prove no effect of the drug; The ethics committee identifies ethical issues in the experimental process; The prespecified interim analysis finds the expected difference in efficacy between the groups.

### 2.6. Randomization, allocation, and blinding

Randomization and allocation will start on day 2 or 3 of the previous menstrual cycle before IVF. Eligible women will be stratified by AFC (<5 or ≥5) and randomized within the 2 strata. All participants will be randomly assigned to either the EZTG group or the placebo group in a 1:1 ratio. Randomization will be completed by software (R 4.0.0, R Foundation for Statistical Computing, Vienna, Austria), and different-sized block groups of 2, 4, 6 will be applied. Random number tables will be provided by statistical professionals not involved in the rest of the study and the number will be sealed into brown envelopes. Then the group allocation will be accomplished by opening envelopes sequentially in the order in which patients were recruited.

A double-blind study design will be applied to enhance the quality of the study evidence. Both medications (EZTG granules and placebo) will be prepared in a manner that they will appear similar in appearance, flavor, and smell. Clinicians and patients will not be able to distinguish the difference between groups. The outcome assessment and data analysis will also be conducted in blinding. When the clinical trial is over, the final blind-break process is performed by authorized researchers.

### 2.7. Interventions

#### 2.7.1. Therapeutic drug preparation and administration.

The experimental drug EZTG will be in the form of granules, obtained from the original EZTG formula mixture after decocting, concentrating, and drying. The granules are produced by the Drug Manufacturing Unit of the Affiliated Hospital of Shandong University of Traditional Chinese Medicine and packaged as 3 g/bag, batch number 01-FZ032-03. Genuine medicinal materials are used in all kinds of traditional Chinese herbs including: Cuscuta chinensis (Tu Si Zi), Ligustrum lucidum (Nv Zhen Zi), Herba Ecliptae (Mo Han Lian), Fructus Lycii (Gou Qi Zi), Angelica sinensis (Dang Gui), Radix Rehmanniae Preparata (Shu Di Huang), Ligusticum wallichii (Chuan Xiong), Paeonia lactiflora (Bai Shao), Rhizoma cyperi (Xiang Fu), Radix Glycyrrhizae Preparata (Zhi Gan Cao). All the dosage is within the safe limit specified in the Chinese Pharmacopoeia.

**Figure 2.**
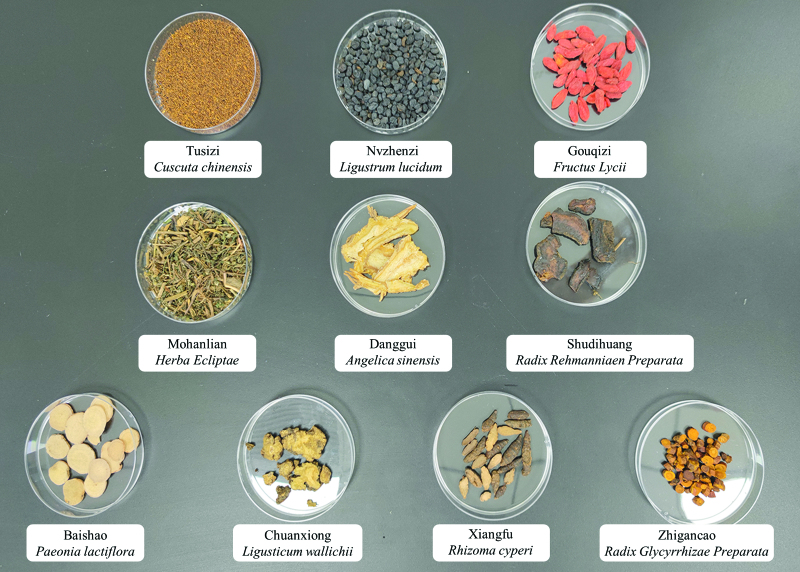
The picture of Chinese herbal medicine in EZTG. EZTG = Erzhi Tiangui.

Method of administration: 3 bags each time, 3 times a day, orally, administered after being dissolved in warm water. Participants were required to take the EZTG from day 2 or 3 of the previous cycle that precedes the IVF cycle to the day of the trigger. The crude herbal dosage and pharmacological efficacy of the herbs in EZTG granules are summarized in Table 2. Figure 2 shows the picture of Chinese herbal medicine in EZTG.

**Table 2:** Ingredients and effects of Chinese herbal medicine in the EZTG granules.

Components	Crude herbal dosage (g/d)	Related pharmacological effects
Tusizi (Cuscuta chinensis)	9	Anti-inflammation, anti-aging, relieving pain, or aphrodisiac.
Nvzhenzi (Ligustrum lucidum)	9	Abrogation of cell apoptosis and cell senescence; hepatoprotective effect, anticancer activity, antioxidant activity, and immunomodulating effect.
Mohanlian (Herba Ecliptae)	9	Promoting osteoblastic differentiation, and thus treating osteoporosis.
Gouqizi (Fructus Lycii)	9	Oxidative stress alleviation, antiaging, and senescence delay.
Danggui (Angelica sinensis)	6	Hematopoietic activity, promoting immunity, antitumor, anti-inflammation, antioxidant, anti-aging, anti-virus, and liver protection.
Shudihuang (Radix Rehmanniaen Preparata)	9	Improvement of energy metabolism dysfunction, relegation of the peripheral circulation system, and relieving oxidative damage in the body.
Chuanxiong (Ligusticum wallichii)	6	Regulation of the circulatory system, anti-inflammation, and abrogation of cell apoptosis.
Baishao (Paeonia lactiflora)	6	Activation of ovarian angiogenesis and follicular development.
Xiangfu (Rhizoma cyperi)	6	Anti-depression, hypoglycemic effect, anti-oxidation, anti-inflammation, anti-tumor and antibacterial effect.
Zhigancao (Radix Glycyrrhizae Preparata)	10	Regulation of lipid and glucose metabolisms and anti-cancer, as well as immunomodulatory and hepatoprotective effects.

EZTG = Erzhi Tiangui.

#### 2.7.2. Placebo preparation and administration.

The placebo granules, which were mainly composed of dextrin and caramel, were made in a similar color and shape to EZTG. The placebo granules have no active ingredients and therefore have no efficacy. The method of administration was the same as the EZTG. Both the EZTG and placebo production will be consistent with the Chinese Medicine Standards of the State Food and Drug Administration.

#### 2.7.3. COS and oocytes retrieval.

Participants will be assessed again for eligibility on day 2 or 3 of the IVF cycle. The participants will also receive hormonal and ultrasound assessments to ensure the feasibility of COS.

Eligible participants will be administrated COS with a Gonadotrophin-releasing hormone antagonist protocol on day 2 or 3 of the menstrual cycle and given recombinant follicle stimulating hormone (Gonal-F, Merck Serono, Switzerland). The starting dose of rFSH will be in a range between 150–225 IU/day, based on the individual baseline characteristics. The COS process will be individualized, and the follow-up dose of rFSH will be adjusted according to the ovarian response during COS every 2 or 3 days. On day 5 or 6 of the ovarian stimulation, gonadotrophin releasing hormone-antagonist (cetrorelix acetate, Cetrotide; Merck Serono, Germany) will be initiated. When more than 2 follicles are at least 18 mm, 250 mg of recombinant human chorionic gonadotrophin will be administered to trigger the final follicular maturation. After 35 to 37 hours, transvaginal ultrasound-guided aspiration is performed. Intracytoplasmic sperm injection will only be performed in cases of severe male factors of infertility.

#### 2.7.4. Embryo transfer and luteal phase support (LPS).

All patients received cleavage-stage embryo transfer on day 3 after oocyte retrieval, or blastocyst transfer on day 5, except for the following cases: uterine or endometrial abnormalities, such as endometriosis, uterine fibroids, endometrial polyps or intrauterine adhesions; serum *P* > 1.5 ng/mL on the trigger day. No more than 2 embryos will be transferred per transfer. Among participants receiving embryo transfer, LPS will be administrated until 10 weeks of gestation, with an intramuscular P4 injection of 40 mg per day or vaginal P4 (8% Crinone, Merck Serono, Switzerland) 90 mg per day, plus dydrogesterone tablet (Duphaston, Abbott, Hoofddorp, Netherlands) 30 mg per day. The LPS will be discontinued if the pregnancy failure has been confirmed by the serum pregnancy test or ultrasound assessment.

### 2.8. Outcomes

#### 2.8.1. Efficacy outcomes.

The primary outcome is the number of oocytes retrieved per COS cycle.

The following secondary outcomes will also be evaluated as secondary outcomes:

1. Symptom rating scores of kidney qi-yin deficiency syndrome after treatment (The symptom rating scales is shown in Table 3).
2. Difference in rating scores of kidney qi-yin deficiency syndrome before and after treatment.
3. Average daily dose of gonadotropin per COS cycle.
4. Total gonadotropin dose and duration of ovarian stimulation per COS cycle.
5. Cycle cancelation rate per COS cycle. Cycle cancelation is defined as a cycle canceled before obtaining at least 1 viable embryo for any reason.
6. Good quality embryos rate per embryo. A good quality embryo is defined as an embryo that is graded as 6-cell grade 2 (6CII) or better or blastocyst.
7. Embryo implantation rate per embryo, defined as the number of gestation sacs detected divided by the number of embryos transferred.
8. Clinical pregnancy rate per patient, defined as the number of women with identified clinical pregnancies divided by the number of patients randomized to a specific group. Clinical pregnancy is confirmed when 1 or more gestational sacs are detected on transvaginal ultrasound assessment.
9. Early miscarriage rate per patient, defined as the percentage of participants with loss of a diagnosed clinical pregnancy before 12 weeks gestation to the total patients randomized.
10. CLBR per patient, defined as the proportion of deliveries with at least 1 live birth per started cycle or oocyte aspiration, including all fresh and/or frozen embryo transfers until 1 delivery with a live birth or until all embryos were used (within 1 year after randomization).

**Table 3:** Symptom rating scales of kidney qi-yin deficiency syndrome.

Symptoms	Characteristics and severity of the symptoms	Score
The color and quality of the menstruation	The color of the blood is dark red and the quality is thin and watery	5
	The color of the blood is dark red or the quality is thin and watery	3
	Normal color and quality	0
The waist and knees are sour and powerless, or pain in the heels	Severe discomfort that interferes with daily life	6
	Moderate discomfort	4
	Mild discomfort	2
	No obvious discomfort	0
Dizziness and tinnitus	Severe, occurs constantly and interferes with daily life	6
	Moderate, occurs frequently	4
	Mild, occurs occasionally	2
	Not obvious	0
Mental exhaustion	Severe, occurs constantly and interferes with daily life	6
	Moderate, occurs frequently	4
	Mild, occurs occasionally	2
	Not obvious	0
Low sexual desire	Low sexual desire, no intercourse	6
	Low sexual desire, intercourse once a month	4
	Low sexual desire, intercourse once a week	2
	Normal sexual desire	0
Less vaginal discharge, and loss of armpit or pubic hair	Less vaginal discharge and loss of armpit or pubic hair	6
	Less vaginal discharge or loss of armpit or pubic hair	4
	Thin armpit or pubic hair	2
	Normal	0

#### 2.8.2. Safety outcomes.

Data on vital signs and relevant laboratory tests including blood routine tests, urine routine tests, liver and kidney function tests, fecal tests, and electrocardiography, will be collected at baseline assessment and on the day of the hCG trigger. The adverse events will be recorded and evaluated at every visit of participants during the study period.

### 2.9. Statistical analysis

#### 2.9.1. Sample size calculation.

##### 2.9.1.1. Sample size calculation

Sample size calculations referred to previous studies, and the clinical outcomes of aged women with POR from the electronic database in our reproductive center.^[9,23]^ The study was designed to detect a difference between groups in terms of 1 additional retrieved oocyte after the hCG trigger with a standard deviation of 3 retrieved oocytes. The power is set at 90%, along with a 2-sided 5% type I error rate. To account for a 20% dropout rate, the sample size of 480 was determined using PASS 15.0 (NCSS, LLC, Kaysville, Utah), in which 240 participants were in each group.

#### 2.9.2. Planned data analysis.

Intention-to-treat (ITT) analysis will be used for baseline comparisons and the assessment of the effect of EZTG on the total number of retrieved oocytes per COS cycle, as well as other secondary outcomes. T-test, Wilcoxon rank-sum test, or chi-square test will be used to test for differences. The results will be given as mean (standard deviation), median (interquartile range), or frequency (percentage) according to the type and distribution of the variables. A 2-sided *P* < .05 is considered statistically significant with a confidence interval of 95%. Per protocol analysis will be conducted as a sensitivity analysis, including those patients who completed their randomized treatment. If the ITT results conflict with the per protocol results, we will interpret the study results according to the outcome of the ITT analysis.

In addition, a subgroup analysis will be performed according to AFC strata (<5 or ≥5) using a similar approach to the primary analysis. Multiple imputations will be used to process missing values in the data of this study. All statistical work will be performed on SPSS version 26.0 software (IBM Corporation, Armonk, NY).

### 2.10. Data management

All the data collected in the baseline screening, or at time points during the study period, will be recorded on the day participant visit the clinic. The embryological-related data will be recorded each day from the oocyte retrieval to the embryo transfer. Follow-up in the study will continue until 1 year after randomization, or during which time a live birth is achieved or all embryos are transferred.

Researchers at each reproductive center will undergo data management training to ensure the data reliability and privacy of participants. The data will be collected in compliance with the prespecified standard operating procedure. Collected data will be uniformly managed by the database of the Reproductive Center of Shandong University of Traditional Chinese Medicine. All protocol deviations or modification results should be recorded in detail and properly documented. The data will be collected, entered, and coded into a standard case report form (CRF) using the double-entry method. CRFs will be kept in locked filing cabinets. And the electronic CRFs will be kept in computers with passwords and access will only be allowed to the principal researchers. All data will be de-identified by CRFs and will be kept for 5 years. After that period, the electronic data will be removed.

### 2.11. Trial monitoring

A trial monitoring committee (TMC) has been established, including principal researchers in each reproductive center. The TMC is responsible for data monitoring, adverse event assessment, regulation of the study process monthly, the interim analysis, and the decision on terminating the study or not.

### 2.12. Ethics and consent to participate

The ethical approvals of the study have been obtained from all participating reproductive centers. EZTG formula has been used in some reproductive centers as one of the conventional treatments for infertility and applied as a complementary treatment in ART. So far, there are no adverse events related to EZTG reported. In the study, all of the medications used in IVF cycles are regular and comply with the routines. The intensity of adverse events will be categorized as light, moderate, serious, and suspected unexpected serious adverse events. Serious adverse events should be reported to the TMC immediately. Meanwhile, the blind-break process will be performed, and the trials will be discontinued. If the relationship with the trial is confirmed, we will compensate the participants accordingly according to the “Regulation on the Handling of Medical Malpractice” in China. Written informed consent is necessary from each couple as a prerequisite for the trial.

## 3. Discussion

The decline of fertility in advanced-age women can be attributed to 2 aspects: 1 is the ovarian reserve diminishes with aging. The second is the decline in the quality of oocytes, which subsequently results in poor-quality embryos.^[24]^ During the COS process in IVF-ET, this population usually demonstrates POR to exogenous gonadotrophin. And the oocytes retrieved under transvaginal ultrasound-guided aspiration are relatively fewer, which directly leads to poor live birth outcomes.^[25]^ The treatment strategies for advanced-age women with POR can be summarized as “one more oocyte matters,”^[11]^ and this explains why we apply “the number of retrieved oocytes” as the primary outcome in our study.

An important part of TCM theory is the “holistic concept,” which means the TCM treatment usually involves multi-system, multi-pathway, and multi-target. Therefore, the current studies on the mechanism of EZTG efficacy also include several hypotheses. Two studies reported that EZTG can reduce the apoptosis of granulosa cells by inhibiting the PT3K/Akt1 and Fas/FasL signaling pathways.^[15,16]^ The level of apoptosis of granulosa cells is proven to be closely associated with the quantity and quality of oocytes because of the complex connection between granulosa cells and the oocytes, which regulates the recruitment, development, maturation, and atresia of the oocytes.^[26,27]^

This effect of EZTG can also be achieved by promoting the expression of the mitochondrial membrane protein Mitofusin 2 to regulate the energy metabolism of granulosa cells.^[17]^ Sun et al also reported many of the differential proteins associated with apoptotic and proliferative processes in the follicular fluid between EZTG and placebo groups.^[18]^

In addition, Glial cell line-derived neurotrophic factor and its receptors have been found to express in the human ovary and have crosstalk with the reproductive endocrine signaling system, which indicates its involvement in the regulation of primordial follicular activation.^[28,29]^ A previous mouse-model study reported that EZTG promoted the expression of receptor of GDNF, facilitated the expulsion of the first polar body, and improve the quality of oocytes and embryos.^[19]^

We previously conducted a pilot study, in which 100 women of advanced age undergoing IVF were included and allocated to EZTG or placebo groups in a 1:1 ratio.^[18]^ However, among clinical outcomes, no statistical difference existed except for the high-quality embryos were more after giving EZTG. In comparison with the pilot study, this study design has the following optimizations: Firstly, the pilot study was a single-center design with a limited sample size of 100 women. Our study will recruit 480 women from 10 reproductive centers, which is more reflective of real-world conditions. And the sample size is calculated according to the prespecified primary outcome to ensure the power to detect the difference between groups. Secondly, the pilot study focused on women of advanced age, while the ovarian reserve and response were not considered, which made the study population relatively large and resulted in confounding bias. In our study, we include a more homogeneous and comparable study population of advanced-age women with POR, defined by ESHRE Bologna criteria. Thirdly, we prolong the follow-up period to 1 year after randomization and evaluated CLBR per patient. Symptom rating scores of kidney qi-yin syndrome are also evaluated because the participant perception of the symptoms is pivotal according to TCM theory. In addition, we advanced and prolonged the treatment period of EZTG, because we expected the pretreatment to be beneficial to the recruitment of follicles before COS. Our study has also some limitations. The study had the power of 90% to detect a difference of 3 retrieved oocytes between the 2 groups. Therefore, smaller but clinically important differences might be overlooked, and the error is more likely to occur in subgroup analysis due to decreased sample size.

In conclusion, the results of this study are expected to provide high-quality evidence for the effectiveness and safety of EZTG formula as an adjuvant treatment in IVF-ET for advanced-age women with POR.

## Acknowledgments

The authors would like to thank all the participants and researchers for their contributions to this study.

## Author contributions

**Conceptualization:** Jing-Yan Song, Zhen-Gao Sun.

**Investigation:** Qiao-Song Han, Jing-Yan Song, Yue Zhou.

**Methodology:** Jing-Yan Song, Wen Chen, Yue Zhou.

**Project administration:** Wen Chen.

**Writing – original draft:** Qiao-Song Han.

**Writing – review & editing:** Jing-Yan Song, Zhen-Gao Sun.
